# Surgery

**Published:** 1898-08

**Authors:** John Parmenter, Nelson W. Wilson

**Affiliations:** Buffalo, N. Y.; Professor of anatomy and adjunct professor of clinical surgery, Buffalo University Medical College; Buffalo, N. Y.


### Progress in Medical Science.

### SURGERY.

CONDUCTED BY JOHN PARMENTER, M. D., Buffalo, N. Y.
Professor of anatomy and adjunct professor of clinical surgery, Buffalo University Medical College.
AND
NELSON W. WILSON, M. D., Buffalo, N. Y.

### DIGEST OF SURGICAL MATTERS DISCUSSED AT THE DENVER MEETING OF THE AMERICAN MEDICAL ASSOCIATION.

### SURGERY OF THE LUNG.

DR. JOHN B. MURPHY, of Chicago, delivered an address on The surgery of the lung. Dangers of pneumotomy are hemorrhage from chest openings; pneumothorax from bronchial openings; pleuritis of both sides; traumatic pneumonia and sepsis, dyspnea and shock. Pathological conditions requiring excision are hernia, infected injuries, abscesses, bronchiectasis, gangrene, tuberculous cavities, foreign bodies, hydatids and actinomycosis. In neoplasms of the chest wall involving the lungs excision is desirable. Lungs may be defunctionalised and quiescence maintained by pleural injections, rib resections and thoracotomy with separation of pleural adhesions.

Adhesions and consolidations are technical difficulties of intrapleural injection. The dangers are given as air embolism, subpleural emphysema, pulmonary emphysema, dyspnea and sepsis. Shock, hemorrhage, sepsis and pneumothorax are the dangers of operations on the chest wall.

The dyspnea of pneumothorax, Dr. Murphy holds, is caused by the vibration of the mediastinal septum nullifying the piston action of the diaphragm. Immobilisation of the septum lessens the danger. Pulmonary tuberculosis was cured by surrounding the focus of infection by connective tissue; this tissue growth is favored by immobilisation.

For compressing the lung Dr. Murphy has injected nitrogen gas, which is least active and slowly absorbed. He injected 140 cubic inches in one case, with marked relief to the patient.

There is no danger of dyspnea so long as the chest is not opened and the vibration of the diaphragm is prevented. There is, however, possibility of air embolism by reason of injury to a vein by the trochar. This treatment is of benefit in early tuberculosis where there is a small superficial cavity in the apex.

### INFLUENCE OF AGE, SEX AND RACE IN SURGICAL AFFECTIONS.

In his address on the Influence of age, sex and race in surgical affections, Dr. W. L. Rodman, of Louisville, presented some interesting facts. Malignant disease is increasing in the black in greater ratio than in the white race; the negro is more prone to sarcoma than to cancer; rectal cancer is relatively common. Local tuberculosis of joints, lymphatics and serous membranes, show marked increase in the negro race. Varicocele is uncommon in the negro, because the scrotum fits more closely and supports the veins better. In 600 Louisville cases one was a mulatto. Full-blooded negroes are not affected Enlarged prostate is rarely seen in the negro, but is common among whites. Gall-stones are more common in women, rare in negroes; rare after thirty years. Aneurism is over three times as frequent in negroes as in whites; three times more frequent in males. Tetanus is more common in hot climates among dark-skinned persons; highest death-rate between five and fifteen years, five times higher in boys. Tetanus increases in army life following defeats and variations of temperature. Ovarian tumors occurred at all ages and in every race; cystic and dermoid most common; less frequent in negresses. Malignant ovarian disease most frequent before the age of fifteen.

### AN INCISION FOR BILE TRACT OPERATIONS.

Dr. A. D. Bevan, of Chicago, described an incision suitable for exploration or extensive operation on the bile tract. It consists of an outer part like an italic S through the outer border of the rectus, three or four inches long. To this is added extended portions, one to three inches in length. The extended portions should not, however, completely divide the entire thickness of the rectus. The advantages claimed for this opening are that danger of hernia is lessened and a minimum amount of nerve supply is injured. The incision should not come within three-quarters of an inch of the costal arch.

Dr. John B. Hamilton, of Chicago, locates the gall-bladder by drawing a line from the ensiform cartilage to the superior spine of the ilium and another from the umbilicus to the tenth costal junction. The gall-bladder is found at a point one-half to three-quarters of an inch above the intersection of the lines.

---

INTESTINAL OBSTRUCTION FROM GALL-STONE.

Dr. J. P. LORD, of Omaha, read a paper on Intestinal obstruction from gall-stone, and reported the removal of a stone "large as a hen's egg" from the bowel of a woman 70 years old. Abdominal distension is most marked where the obstruction is in the rectum, sigmoid or some portion of the large intestine. Fifty to 80 per cent. should be saved by operation. Mortality is lowest in early operation. Lavage of the stomach should precede operation. This removes the danger of the patient being choked by vomited material.

---

REMOVAL OF THE STOMACH.

REMOVAL of the stomach occupied considerable attention. Dr. P. S. Connor reported removal of the stomach fifteen years ago. The patient died on the table, hence only a brief report was made of the case at the time. The operation is admissible only in case of carcinoma and when the walls of the organ were so infiltrated as to render the stomach functionally dead.

The question of injury to the pneumogastric nerve was raised. Dr. Harris, of Chicago, replied by reporting a case of entire removal of the stomach and stated that the right pneumogastric nerve passed high up and could be avoided provided one kept in the omentum near the stomach.

---

IN DIAGNOSIS and surgical treatment of malignant obstruction of the pylorus, Dr. W. J. Mayo, of Rochester, Minn., advocated early exploratory operation. Gastro-duodenostomy is preferable to gastro-jejunostomy. The Murphy button is much more successful than sutures. In operating, the stomach wall is cut to the mucous coat before the puckering string is put in, the bite of the suture should not be too deep. Fatal septic pneumonia from aspiration of vomited matter should be guarded against by the anesthetiser.

### INTESTINAL ANASTOMOSIS.

Two new methods of intestinal anastomosis were reported by Dr. W. F. Metcalfe, of Detroit, and Dr. Ernest Laplace, of Philadelphia, at the American Medical Association meeting. Dr. Metcalfe used cylinders of hard sugar; really a modification of the old potato method. Dr. Laplace uses a new forceps, consisting of two rings, which serve as supports during the suturing. These rings are separable into halves, which are withdrawn before the last sutures are placed. This leaves no foreign substance in the intestine.

---

### ANEURISM OF THE AORTIC ARCH.

Dr. B. Merrill Ricketts, of Cincinnati, reported "excellent results" in the treatment of aneurism of the aortic arch by ligation of the right carotid and subclavian arteries.

---

### POPLITEAL ARTERY WOUNDS.

In penetrating wounds of the popliteal artery, Dr. George W. Miel, of Denver, recommended double ligation and extirpation in all cases of incised wounds more than one-eighth of an inch long. Close the external wound loosely.

---

### CLAMPING THE CAROTID.

Dr. George W. Crile, of Cleveland, has demonstrated that the common carotid can be closed by clamp for from twelve to twenty-four hours without injurious results.

---

### CARCINOMA OF THE AXILLA.

Dr. W. D. Graham, of Chicago, concluded an interesting paper on primary carcinoma of the axilla by stating his belief that the origin is in the large tubular convoluted sweat glands; that many cases of fibro-adenoma originate in these glands.

---

### THE SERUM TREATMENT OF SARCOMA.

Dr. William B. Coley, of New York, reported that the use of mixed toxins of erysipelas and bacillus prodigiosus is beneficial in inoperable sarcoma, in all cases save in the rapidly-growing round-cell sarcoma of bone. He reported many cases in which relief had been obtained.

Dr. McArthur, of Chicago, reported a successful treatment, as did Dr. Lewis, of Topeka.

THE CANCER OF THE RECTUM.

Dr. W. W. Keen, of Philadelphia, recommends a permanent artificial anus and total closure of the social end of the rectum in operations for cancer of the rectum. He resects the bowel and treats it as in a lateral anastomosis—invaginating and inserting a row of Lembert's sutures.

TREATMENT OF T-SHAPED FRACTURES.

Dr. Charles A. Powers, of Denver, presented two cases of T-shaped fractures at the elbow joint. He objects to the dressing in extension. His treatment is as follows: Arm slightly abducted and in a position of semi-flexion—140 to 150 degrees—a plaster of Paris bandage is applied. In a week the arm should be flexed a little more and again put up in plaster; in another week the arm put up at right angles and left so for two or three weeks, when all bandages are to be removed.

SUTURING AND BRIDGING DIVIDED NERVES.

The essentials for process in suturing divided nerves are, according to a paper read by Dr. A. H. Levings, of Milwaukee: Absolute asepsis; the nerve should not be separated from its connective tissue surroundings; the nerve should be approximated evenly and without tension; if an inch or more is lost, bridge the gap with material having longitudinal fibers; absolute rest of the limb is not a necessity; the nerve ends should be allowed to just touch each other. If the gap to be bridged is not over three inches long, six strands of silk make an excellent bridge between the ends; muscle is also good bridging material.

ETHER PNEUMONIA.

In a paper on ether pneumonia, Dr. James M. Anders, of Philadelphia, mentions forty-six pneumonias in 57,842 cases reported. He believes the condition is much more frequent. It is not improbable that microörganisms in the upper air-passages are drawn into the lungs during etherisation. A latent or active tuberculous focus of the lungs might, therefore, cause serious trouble.

Prophylaxis consists of properly cleansing the upper air-passages and frequently turning the patient's head to one side. Avoid cooling the body surface and don't waste ether.

